# Characterization of clutch traits and egg production in six chicken breeds

**DOI:** 10.5713/ab.22.0369

**Published:** 2023-01-11

**Authors:** Lei Shi, Yunlei Li, Adam Mani Isa, Hui Ma, Jingwei Yuan, Panlin Wang, Pingzhuang Ge, Yanzhang Gong, Jilan Chen, Yanyan Sun

**Affiliations:** 1 Key Laboratory of Animal (Poultry) Genetics Breeding and Reproduction, Ministry of Agriculture and Rural Affairs, Institute of Animal Science, Chinese Academy of Agricultural Sciences, Beijing 100193, China; 2 College of Animal Science and Technology, Hebei Agricultural University, Baoding 071001, China; 3 Key Laboratory of Agricultural Animal Genetics, Breeding and Reproduction of Ministry of Education, College of Animal Science and Technology, Huazhong Agricultural University, Wuhan 430070, China

**Keywords:** Chicken, Clutch Length, Clutch Trait, Egg Production, Laying Performance

## Abstract

**Objective:**

The better understanding of laying pattern of birds is crucial for developing breed-specific proper breeding scheme and management.

**Methods:**

Daily egg production until 50 wk of age of six chicken breeds including one layer (White Leghorn, WL), three dual-purpose (Rhode Island Red, RIR; Columbian Plymouth Rock, CR; and Barred Plymouth Rock, BR), one synthetic dwarf (DY), and one indigenous (Beijing-You Chicken, BYC) were used to characterize their clutch traits and egg production. The age at first egg, egg number, average and maximum clutch length, pause length, and number of clutches and pauses were calculated accordingly.

**Results:**

The egg number and average clutch length in WL, RIR, CR, and BR were higher than those in DY and BYC (p<0.01). The numbers of clutches and pauses, and pause length in WL, RIR, CR, and BR were lower than those in DY and BYC (p<0.01). The coefficient variations of clutch length in WL, RIR, CR, and BR (57.66%, 66.49%, 64.22%, and 55.35%, respectively) were higher than DY (41.84%) and BYC (36.29%), while the coefficient variations of egg number in WL, RIR, CR, and BR (9.10%, 9.97%, 10.82%, and 9.92%) were lower than DY (15.84%) and BYC (16.85%). The clutch length was positively correlated with egg number (r = 0.51 to 0.66; p<0.01), but not correlated with age at first egg in all breeds.

**Conclusion:**

The six breeds showed significant different clutch and egg production traits. Due to the selection history, the high and median productive layer breeds had higher clutch length than those of the less productive indigenous BYC. The clutch length is a proper selection criterion for further progress in egg production. The age at first egg, which is independent of clutch traits, is especially encouraged to be improved by selection in the BYC breed.

## INTRODUCTION

Egg production is the primary interest for genetic-economic improvement in laying hens. The traditional direct selection of part- or whole-record egg number or egg-laying rate resulted with positive genetic progress in egg production. However, this may reduce the genetic variability, narrow the range of selective response, and slow down further genetic progress [1]. Therefore, it is necessary to explore new and more basic biological traits for further genetic improvement of egg production [2].

Under the 24 h based light-dark period, the pronounced cyclic process of chickens’ egg formation requires an average a bit over 24 h [3–5]. In a sequence of consecutive days, the first oviposition normally occurs in the morning, and the following oviposition is postponed day by day until a laying cessation for one or more days when the oviposition is postponed to the afternoon. Then the next egg will be laid in the morning again in a new sequence. Such a series of eggs laid on successive days was named a clutch [6]. The clutch length, indicating the number of consecutive days with eggs, is an important trait describing the individual laying pattern and has been investigated in different domestic avian species in recent years. The meaningful correlation between clutch length or clutch number with egg production has been reported [7,8]. Moderate heritabilities were estimated for clutch length, such as 0.31 in Rhode Island Red (RIR), 0.34 in White Leghorn (WL), and 0.43 in Dwarf brown-egg layers [1,7]. Clutch traits, providing additional insight into the difference between highly productive and inferior layers, were proposed to be integrated into selection criteria of layer chickens [1,9–12] and other domestic poultry species [13,14]. Increased clutch length could reduce the average follicle growth period, enhance the growth rate, and improve egg production at the end. The hens that produce eggs in long clutches are favored. Chen and Tixier-Boichard [9] reported that 16 generations of selection for average clutch length in two dwarf brown-egg layer lines resulted in an increase from 4 to 15 for egg number per clutch and an increase from 105 to 130 for egg number until 42 wk of age.

There is a rising trend where customers are looking more and more for differentiation in egg color and appearance in their egg consumption. Furthermore, concern is increasing for the welfare of day-old male chicken cull in egg production industry. This inspires the traditional layer or dual-purpose breeding involving a wide variety of distinct breeds or lines [15,16]. The better understanding of the clutch patterns in different types of chickens may provide new insights for their breeding strategy and management practice [14]. However, this information has not been explored in a wide range of breeds. The objective of this study was to analyze clutch traits and egg production in six chicken breeds covering the layer breed, dual-purpose breed, synthetic dwarf line, and indigenous breed, aiming at providing basic evaluation and support for an informed breeding plan.

## MATERIALS AND METHODS

### Ethics approval

The present study was approved by the Animal Care and Use Committee of Institute of Animal Science of Chinese Academy of Agricultural Sciences (No. IAS2021–48) and was performed in accordance with the relevant guidelines and regulations set by Ministry of Agriculture and Rural Affairs of the People’s Republic of China.

### Experimental animals

A total of 4,015 birds from six representative genetic stocks hatched contemporarily were used in this study. These included one layer breed, WL (n = 629); three dual-purpose breeds, RIR (n = 704), Columbian Plymouth Rock (CR; n = 730), and Barred Plymouth Rock (BR; n = 688); one synthetic dwarf line (DY; n = 675), which was a hybrid of Beijing-You chickens (BYC) and Dwarf Jingxing Yellow chickens; and one Chinese indigenous breed, BYC (n = 589). WL, RIR, CR, and BR are pure Shaver lines imported from the University of Guelph, Canada.

### Management

The birds were housed in individual cages of three-tier battery-cage in the same building since 19 wk of age. Artificial light of 10 lx was provided for 8 h per day (8 L:16 D) at the beginning of 19 wk and increased by 1 h per week until 16 h light per day (16 L:8 D) at 27 wk and last until the end of this experiment. The LED lamps were suspended 2 m above the ground. Light intensity was measured at the birds’ eye level with the photoreceptor sensor of a light meter (model: DT-1301; Shenzhen Everbest Machinery Industry Co. Ltd., Shenzhen, China). The house was equipped with thermal controls and was held at around 21°C during the study. Feed and water were provided according to the chicken standard feeding requirement [17]. All birds were fed the same commercial corn-soybean-based diets formulated in mash form. The composition and nutrient levels of the basal diets were exceeded for all six breeds in this study (Table 1).

### Egg production and clutch traits

Egg production from the first egg until 50 wk of age of each individual was recorded by the customized Radio Frequency Identification (RFID)-based data collection system. The age at first egg, egg number, egg-laying rates, average clutch length, maximum clutch length, number of clutches, pause length, number of pauses, and frequency of clutch length (FCL) were calculated based on the egg production data until 50 wk of age for each individual. More specifically, clutch length is the number of eggs laid on successive days of each individual [18]. The pause length is the average length of interval between two clutches. Three FCL categories (≤7 d, 8 to 14 d, and ≥15 d) were calculated. For example, FCL ≤7 d was calculated as follows;


$$\text{FCL }(\leq 7\;\text{d},\%)=\frac{\text{Number of clutch length}\leq 7\;\text{d in an egg production period}}{\text{Number of clutches in an egg production period}}\times 100$$

In addition, average clutch length and maximum clutch length were also calculated based on the egg production data until 32 wk of age for each individual.

### Statistical analysis

In this study, each bird was taken as the experimental unit to obtain parameters. The egg number and clutch traits were analyzed using general linear model procedure of SAS (SAS 9.1, SAS Institute Inc., Cary, NC, USA). The main effect of the model is breed. The percentage data was arcsine transformed before the analysis. Means were compared by Student-Newman-Keuls multiple-range tests when a significant difference was detected. In addition, Pearson’s correlation coefficients (r values) between egg number or age at first egg and clutch traits were calculated. Significance was designated as p<0.05.

## RESULTS

**Figure 1:**
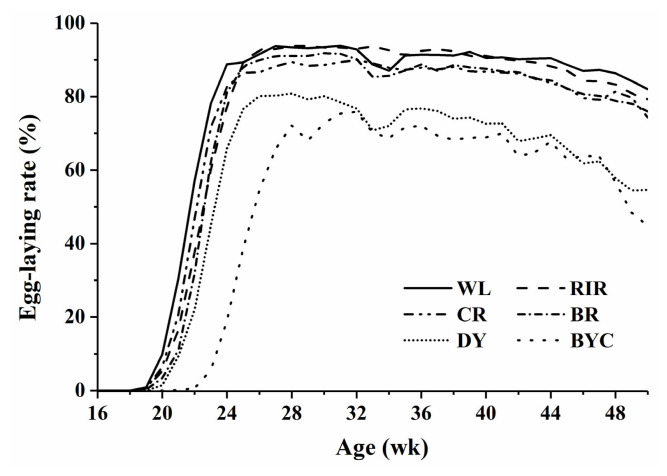
Egg-laying rates curves until 50 wk of age in six chicken breeds. WL, White Leghorn chickens (n = 629); RIR, Rhode Island Red chickens (n = 704); CR, Columbian Plymouth Rock chickens (n = 730); BR, Barred Plymouth Rock chickens (n = 688); DY, synthetic dwarf line (n = 675); BYC, Beijing-You chickens (n = 589).

The six lines showed an overall difference in egg-laying rate. The four breeds WL, RIR, CR, and BR reached their laying peaks between 24 to 27 wk of age, whereas BYC attained the peaks considerably later at 31 wk of age (Figure 1). From 24 wk, the laying rates of WL and RIR breeds were maintained above 80% throughout the experiment, while the laying rates of DY and BYC breeds declined sharply after the laying peaks.

The WL, RIR, CR, and BR breeds had the earlier age at first egg than DY and BYC (p<0.01; Table 2). The most divergent age at first egg was 144.26 d and 170.98 d in WL and BYC, respectively. The egg number and average clutch length in WL, RIR, CR, and BR were higher than those in DY and BYC (p<0.01). Meanwhile, the maximum clutch length in WL and RIR breeds were longer than those in other breeds (p<0.01). The most divergent maximum clutch lengths until 50 wk of age were 48.13 d and 11.11 d in WL and BYC, respectively. The number of clutches and pauses, and pause length in WL, RIR, CR and BR breeds were lower than those in DY and BYC (p<0.01).

More than 90% of the clutch length of DY and BYC were ≤7 d, while 55.68%, 56.60%, 64.45%, and 67.22% in WL, RIR, CR, and BR fell in this category (p<0.01), respectively. Conversely, 24.87%, 25.52%, 13.94%, and 13.04% of clutches were ≥15 d in WL, RIR, CR, and BR breeds, respectively, which were higher than those in DY (2.31%) and BYC (1.44%) (p<0.01).

**Figure 2:**
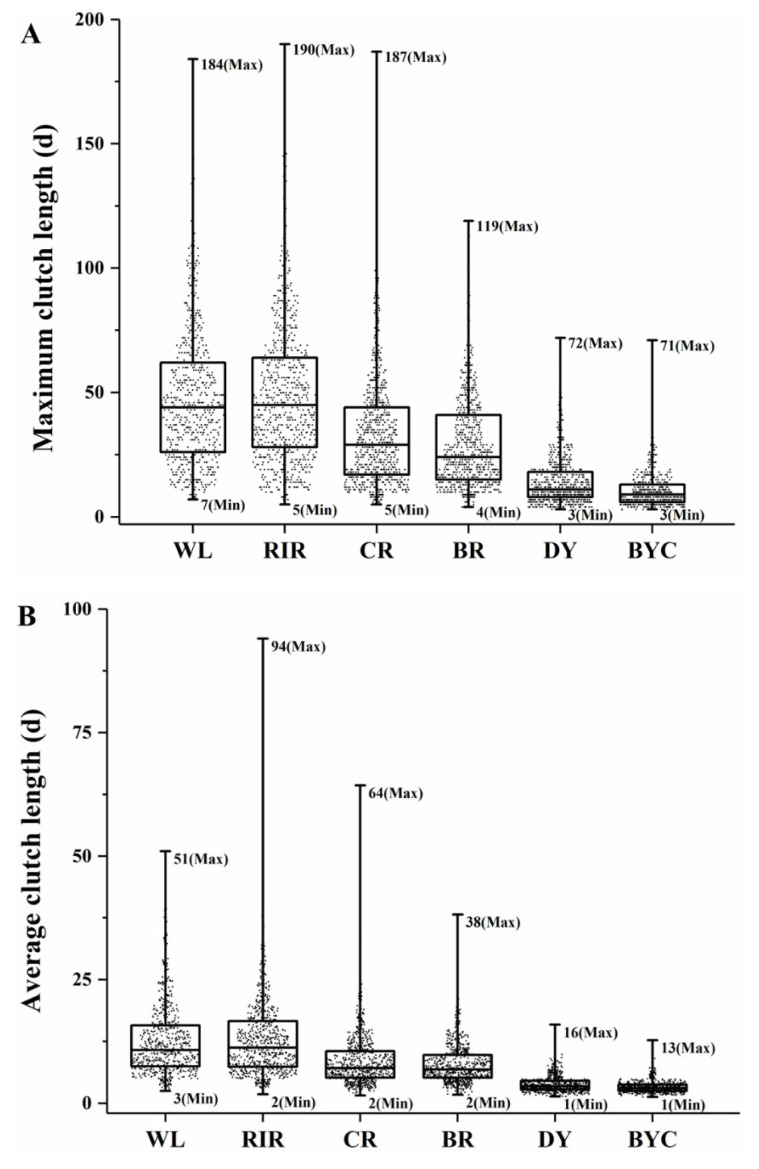
The box plot of maximum (A) and average clutch length (B) in six chicken breeds. The dot indicated maximum clutch length of a chicken. WL, White Leghorn chickens (n = 629); RIR, Rhode Island Red chickens (n = 704); CR, Columbian Plymouth Rock chickens (n = 730); BR, Barred Plymouth Rock chickens (n = 688); DY, synthetic dwarf line (n = 675); BYC, Beijing-You chickens (n = 589).

As shown in Table 3, the coefficient variations of egg number in WL, RIR, CR, and BR breeds (9.10%, 9.97%, 10.82%, and 9.92%, respectively) were lower than those in DY (15.84%) and BYC (16.85%). However, the coefficient of variations of average clutch length in WL, RIR, CR, and BR breeds (57.66%, 66.49%, 64.22%, and 55.35%, respectively) were higher than those in DY (41.84%) and BYC (36.29%). Meanwhile, the interval between upper and lower quartiles of average clutch length and maximum clutch length in WL, RIR, CR, and BR were higher than those in both DY and BYC breeds (Figure 2).

**Figure 3:**
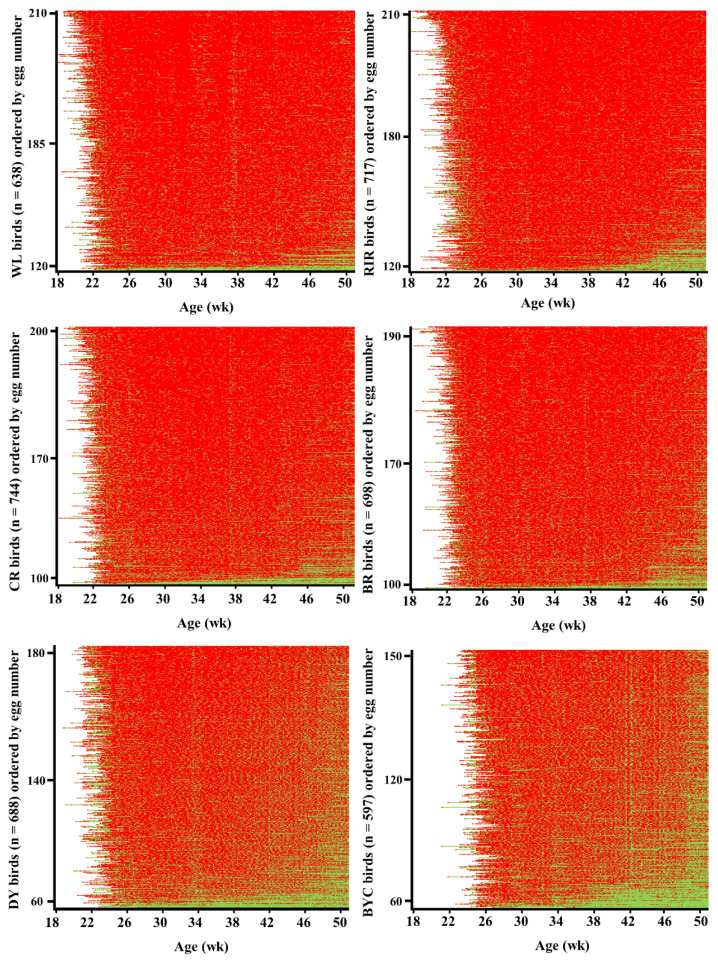
Heatmap showing laying and pause days for individual birds in six chicken breeds until 50 wk of age. The vertical axis represents individuals in the order of egg number. The red dots mean laying days, and the green dots mean pause days. WL, White Leghorn chickens; RIR, Rhode Island Red chickens; CR, Columbian Plymouth Rock chickens; BR, Barred Plymouth Rock chickens; DY, synthetic dwarf line; BYC, Beijing-You chickens.

The laying pattern of individual birds in six chicken breeds are visually shown in Figure 3. A small cluster of hens with less egg number in WL, RIR, CR, and BR showed poor persistence of laying after 46 wk of age. While majority of the DY and BYC chickens exhibited breaks between short clutches.

Correlation coefficients between egg number and clutch traits are shown in Table 4. In all breeds, egg number was positively correlated with average clutch length (*r* = 0.51 to 0.66; p<0.01), maximum clutch length until 32 wk and 50 wk (*r* = 0.41 to 0.54; p<0.01), and FCL ≥15 d (*r* = 0.46 to 0.59; p<0.01). Egg number was negatively correlated with pause length in all breeds (p<0.01). Egg number was also negatively correlated with the number of clutches and number of pauses (p<0.01) in all breeds except for BYC. As shown in Table 5, the age at first egg was negatively correlated with egg number in all breeds (*r* = −0.31 to −0.52; p<0.01) but did not show strong correlations with clutch traits (Table 5).

## DISCUSSION

Eggs are the most abundant animal products in livestock production and the principal protein resources in human diets [19]. Productive laying hens are the everlasting aim of selective breeding practices in the industry. In the present study, the egg production and clutch related traits were investigated in six distinct breeds to better understand the laying pattern for developing informed breeding schemes and management practices designed for different breeds.

The six breeds in this study did show substantial differences in their clutch traits and egg production traits. The indigenous BYC started to lay as late as 170 d. The WL, RIR, CR, and BR had longer clutch length than BYC and synthetic dwarf DY. The maximum clutch length in WL and RIR were longer than those in other breeds. Specifically, the longest value of maximum clutch length in WL (184 d) and RIR (190 d) breeds was about 2.5-fold higher than in DY (72 d) and BYC (71 d). The clutch traits are not comparable between studies, since the egg production records used to calculate the clutch traits varied in target ages in different studies [9,20]. Individual recording of daily egg laying revealed that majority of the DY and BYC chickens exhibited more breaks between short clutches, especially during the late laying period. This might be one of the reasons for their low mean egg production. Their difference in clutch traits and egg production traits indicated that differential breeding plans and management should be applied.

Population variation is one of the crucial determinants of genetic progress. It is expected that intense selection of egg number has eroded a high proportion of the genetic variability in components associated. As shown in this study, the coefficient variations of egg number in WL, RIR, CR, and BR breeds (9.10% to 10.82%) were lower than those in DY (15.84%) and BYC (16.85%). This should be due to the generations of selection for egg numbers in WL, RIR, CR and BR breeds. Interestingly, the coefficient variations of average clutch length (55.35% to 66.49%) and maximum clutch length (58.09% to 65.80%) in these four breeds were higher. Positive correlations between egg number, average clutch length, maximum clutch length until 50 wk of age were detected. Therefore, the clutch length seems to be a better selection criterion than the egg number in view of population variation. Furthermore, average, and maximum clutch length until 32 wk of age were also positively correlated with egg number until 50 wk of age. This highlights the possibility that early clutch traits calculated from egg production data recording to an earlier target age could be applied to facilitate the early selection in both high productive and less productive chicken breeds or lines. It is interesting to notice that egg number was highly correlated with the number of clutches/pauses in high productive WL, RIR, and BR breeds, and moderately correlated in the less productive DY, but not correlated in the least productive BYC breed. It is possible that the BYC birds with more clutches having shorter clutch length and the ones with less clutches having longer pause length may both result in lower egg production. The number of clutches/pauses is therefore not a suitable selection criterion for egg production.

Dissecting egg production as a comprehensive phenotype could also provide detailed insights to elucidate specific factors affecting egg production variability. Understanding the relationship between traits of interest, thereby harnessing those relationships to maximize overall response to selection, is necessary to achieving overall breeding objectives. Except for clutch traits, age at first egg is also an important contributor of total egg production [21]. In the present study, the age at first egg negatively correlated with egg number in all six breeds but did not show strong correlations with clutch traits. Similar findings were also reported previously [7,22,23]. These data showed that the selection of age at first egg may increase the egg number without affecting the clutch traits. The age at first egg and clutch traits can be both integrated into the selection criteria. This is especially important for the BYC breed, which showed an extreme late age at first egg. From the view of management practice, Renema et al [24], Zuidhof et al [25], and Shi et al [26] reported that age at first egg was affected by the age of the initiation of photostimulation, while the clutch length was not. The six breeds here were housed in the same environment in this study. This indicates that the light stimulation should be breed specific to properly adapt the body maturation and sexual maturation. Egg quality is also crucial for egg production efficiency. There might be concern regarding the potential negative correlation between egg quality traits and clutch traits [27]. Although the individual egg quality was not estimated in the present study, our previous publication did not observe antagonism between clutch and egg quality traits [22].

In summary, our study investigated clutch and egg production traits in six chicken breeds covering the layer breed, dual-purpose breed, synthetic dwarf line, and indigenous breed. It identified the high and median productive layer and dual-purpose breeds had higher clutch length than those of the less productive indigenous breeds. In addition, the clutch length may be a potential trait for selection to increase egg production further due to its greater variability than traditional part- or whole-record egg number. Furthermore, the clutch length calculated from the egg production recording until 32 wk of age was also correlated with the whole-period egg number and exerted its advantage for early selection. Different from egg number, the age at first egg showed no correlation with clutch traits. It is therefore encouraged to be incorporated as another selection criteria especially for the late maturated and less productive indigenous breed. This study provided basic evaluation of different breed in the aspect of egg production and laying patterns, and the results may support further informed breeding plans and fine management.

## Figures and Tables

**Table 1:** Composition and nutrient levels of the basal diet

Items	From 19 wk of age to 5% egg-laying rate	From 5% egg-laying rate to 50 wk of age
Ingredients (%)		
Corn	65.5	64.0
Soybean meal	21.5	23.2
Wheat bran	5.0	3.8
Limestone	4	5
Premix^1)^	4	4
Formulated nutrient profile^2)^		
ME (MJ/kg)	11.20	11.08
Crude protein (%)	15.07	15.51
Ca (%)	2.03	2.75
Total phosphorus (%)	0.51	0.51
Available phosphorus (%)	0.29	0.29
Lys (%)	0.75	0.78
Met (%)	0.34	0.39
Met+Cys (%)	0.65	0.73

1) Premix provided per kilogram of diet: vitamin A, 100 to 250 KIU; vitamin D_3_, 60 to 80 KIU; vitamin E, 0.5 KIU; vitamin K_3_, 80 mg; vitamin B_1_, 45 mg; vitamin B_2_, 180 mg; vitamin B_6_, 100 mg; vitamin B_12_, 0.5 mg; D-Calcium pantothenate, 220 mg; nicotinamide, 720 mg; folic acid, 20 mg; biotin, 2 mg; Copper, 0.2 to 0.8 g; ferrous iron, 1.5 to 5.0 g; zinc, 0.8 to 2.4 g; manganese, 1.5 to 3.0 g; iodine, 10 to 30 mg; selenium, 2 to 6 mg.

2) The nutrient levels were calculated from the data provided by Feed Database in China.

**Table 2:** Egg production and clutch traits in six chicken breeds

Variables^2)^	Breeds^1)^						SEM	p-value

	WL	RIR	CR	BR	DY	BYC		
Age at first egg (d)	144.26^f^	149.29^d^	147.50^e^	150.32^c^	154.61^b^	170.98^a^	0.20	<0.01
Egg number (n)	180.81^a^	175.52^b^	169.53^c^	165.43^d^	137.28^e^	116.48^f^	0.46	<0.01
Average clutch length until 32 wk (d)	13.36^a^	10.43^b^	8.33^c^	8.20^c^	5.35^d^	4.22^e^	0.11	<0.01
Average clutch length (d)	12.83^a^	13.20^a^	8.65^b^	8.00^c^	3.84^d^	3.31^d^	0.11	<0.01
Maximum clutch length until 32 wk (d)	35.10^a^	28.85^b^	23.19^c^	22.15^c^	13.88^d^	5.35^e^	0.25	<0.01
Maximum clutch length (d)	48.13^a^	49.36^a^	33.29^b^	30.07^c^	14.48^d^	11.11^e^	0.41	<0.01
Number of clutches (n)	18.12^d^	17.80^d^	24.67^c^	25.06^c^	38.75^a^	37.57^b^	0.20	<0.01
Average pause length (d)	1.46^c^	1.51^bc^	1.43^c^	1.43^c^	1.58^b^	1.82^a^	0.04	<0.01
Number of pauses (n)	17.07^d^	16.67^d^	23.78^c^	24.10^c^	38.19^a^	36.84^b^	0.20	<0.01
FCL (≤7 d, %)	55.68^e^	56.60^e^	64.45^d^	67.22^c^	91.05^b^	94.60^a^	0.35	<0.01
FCL (8 to 14 d, %)	19.45^a^	17.88^b^	17.53^b^	19.74^a^	6.64^c^	4.31^d^	0.19	<0.01
FCL (≥15 d, %)	24.87^a^	25.52^a^	13.94^b^	13.04^b^	2.31^c^	1.44^c^	0.26	<0.01

SEM, standard error of the mean; FCL, frequency of clutch length.

1) WL, White Leghorn chickens (n = 629); RIR, Rhode Island Red chickens (n = 704); CR, Columbian Plymouth Rock chickens (n = 730); BR, Barred Plymouth Rock chickens (n = 688); DY, synthetic dwarf line (n = 675); BYC, Beijing-You chickens (n = 589).

2) Unless otherwise stated, the variables were calculated based on the egg production data until 50 wk of age.

a–f Values within a row with different superscripts differ significantly at p<0.05.

**Table 3:** Coefficient of variation (%) of egg number, average clutch length, and maximum clutch length in six chicken breeds

Variables^2)^	Breeds^1)^					

	WL	RIR	CR	BR	DY	BYC
Egg number (n)	9.10	9.97	10.82	9.92	15.84	16.85
Average clutch length until 32 wk (d)	78.25	65.44	68.81	64.88	70.40	56.83
Average clutch length (d)	57.66	66.49	64.22	55.35	41.84	36.29
Maximum clutch length until 32 wk (d)	53.52	50.00	68.81	64.88	70.40	72.47
Maximum clutch length (d)	58.09	58.78	65.46	65.80	72.55	73.05

1) WL, White Leghorn chickens (n = 629); RIR, Rhode Island Red chickens (n = 704); CR, Columbian Plymouth Rock chickens (n = 730); BR, Barred Plymouth Rock chickens (n = 688); DY, synthetic dwarf line (n = 675); BYC, Beijing-You chickens (n = 589).

2) Unless otherwise stated, the variables were calculated based on the egg production data until 50 wk of age.

**Table 4:** Pearson’s correlation coefficients between egg number and clutch traits in six chicken breeds

Variables^2)^	Breeds^1)^					

	WL	RIR	CR	BR	DY	BYC
Average clutch length until 32 wk (d)	0.35^**^	0.31^**^	0.52^**^	0.50^**^	0.48^**^	0.39^**^
Average clutch length (d)	0.59^**^	0.51^**^	0.59^**^	0.60^**^	0.66^**^	0.54^**^
Maximum clutch length until 32 wk (d)	0.52^**^	0.42^**^	0.46^**^	0.45^**^	0.42^**^	0.43^**^
Maximum clutch length (d)	0.54^**^	0.48^**^	0.54^**^	0.54^**^	0.49^**^	0.41^**^
Number of clutches (n)	−0.61^**^	−0.56^**^	−0.65^**^	−0.60^**^	−0.35^**^	−0.04
Average pause length (d)	−0.40^**^	−0.49^**^	−0.42^**^	−0.46^**^	−0.63^**^	−0.64^**^
Number of pauses (n)	−0.61^**^	−0.56^**^	−0.65^**^	−0.60^**^	−0.35^**^	−0.04
FCL (≤7 d, %)	−0.58^**^	−0.54^**^	−0.69^**^	−0.63^**^	−0.57^**^	−0.36^**^
FCL (8 to 14 d, %)	0.02	0.01	0.31^**^	0.31^**^	0.51^**^	0.32^**^
FCL (≥15 d, %)	0.59^**^	0.53^**^	0.59^**^	0.56^**^	0.46^**^	−0.04

FCL, frequency of clutch length.

1) WL, White Leghorn chickens (n = 629); RIR, Rhode Island Red chickens (n = 704); CR, Columbian Plymouth Rock chickens (n = 730); BR, Barred Plymouth Rock chickens (n = 688); DY, synthetic dwarf line (n = 675); BYC, Beijing-You chickens (n = 589).

2) Unless otherwise stated, the variables were calculated based on the egg production data until 50 wk of age.

** p<0.01.

**Table 5:** Pearson’s correlation coefficients between age at first egg and clutch traits in six chicken breeds

Variables^2)^	Breeds^1)^					

	WL	RIR	CR	BR	DY	BYC
Egg number (n)	−0.52^**^	−0.45^**^	−0.40^**^	−0.36^**^	−0.35^**^	−0.31^**^
Average clutch length until 32 wk (d)	−0.20^**^	−0.16^**^	−0.11	−0.12^**^	−0.15^**^	−0.09^**^
Average clutch length (d)	−0.04	0.06	0.03^**^	−0.08^*^	−0.05	−0.01
Maximum clutch length until 32 wk (d)	−0.06	0.05	0.05	0.03	0.01	−0.02
Maximum clutch length (d)	−0.10^*^	−0.06	−0.05	0.01	−0.15^**^	−0.05
Number of clutches (n)	−0.01	−0.10^*^	−0.08^*^	−0.11^**^	−0.12^**^	−0.21^**^
Average pause length (d)	−0.02	0.00	−0.01	−0.06	−0.20^**^	−0.20^**^
Number of pauses (n)	0.01	−0.10^*^	−0.08^*^	−0.11^**^	−0.12^**^	−0.21^**^
FCL (≤7 d, %)	0.02	−0.08^*^	−0.00	−0.04	0.05	−0.02
FCL (8 to 14 d, %)	0.04	0.00	−0.03	0.00	−0.05	0.03
FCL (≥15 d, %)	−0.05	0.08^*^	−0.01	0.06	−0.05	−0.05

FCL, frequency of clutch length.

1) WL, White Leghorn chickens (n = 629); RIR, Rhode Island Red chickens (n = 704); CR, Columbian Plymouth Rock chickens (n = 730); BR, Barred Plymouth Rock chickens (n = 688); DY, synthetic dwarf line (n = 675); BYC, Beijing-You chickens (n = 589).

2) Unless otherwise stated, the variables were calculated based on the egg production data until 50 wk of age.

* p<0.05;

** p<0.01.

## References

[1] Wolc A, Jankowski T, Arango J (2019). Investigating the genetic determination of clutch traits in laying hens. Poult Sci.

[2] Barton NH, Keightley PD (2002). Understanding quantitative genetic variation. Nat Rev Genet.

[3] Zhang ZC, Du XX, Lai S (2022). A transcriptome analysis for 24-hour continuous sampled uterus reveals circadian regulation of the key pathways involved in eggshell formation of chicken. Poult Sci.

[4] Moore RY, Speh JC, Leak RK (2002). Suprachiasmatic nucleus organization. Cell Tissue Res.

[5] Zhao XZ, Gao GL, Wang HW (2017). Effect of photoperiod on serum hormone concentrations during the annual reproductive cycle in geese. Genet Mol Res.

[6] Silver R (1986). Circadian and interval timing mechanisms in the ovulatory cycle of the hen. Poult Sci.

[7] Chen CF, Shiue YL, Yen CJ, Tang PC, Chang HC, Lee YP (2007). Laying traits and underlying transcripts, expressed in the hypothalamus and pituitary gland, that were associated with egg production variability in chickens. Theriogenology.

[8] Isa AM, Sun YY, Shi L (2020). Hybrids generated by crossing elite laying chickens exhibited heterosis for clutch and egg quality traits. Poult Sci.

[9] Chen C, Tixier-Boichard ML (2003). Correlated responses to long-term selection for clutch length in dwarf brown-egg layers carrying or not carrying the naked neck gene. Poult Sci.

[10] Chen C, Tixier-Boichard ML (2003). Estimation of genetic variability and selection response for clutch length in dwarf brown-egg layers carrying or not the naked neck gene. Genet Sel Evol.

[11] Roy BG, Kataria MC, Roy U (2014). Study of oviposition pattern and clutch traits in a White Leghorn (WL) layer population. IOSR-J Agric Vet Sci.

[12] Noda K, Kino K, Miyakawa H, Banba H, Umezawa Y (2002). Persistency of laying strain building by index selection including oviposition time as selection trait in laying hen. J Poult Sci.

[13] Emamgholi BH, Wood BJ, Abdalla EA (2019). Genetic parameters for clutch and broodiness traits in turkeys (Meleagris Gallopavo) and their relationship with body weight and egg production. Poult Sci.

[14] Bao Q, Yao Y, Weng KQ (2022). Research Note: Comparison on laying behavior and clutch traits among Zhedong white geese (Anser cygnoides), Sichuan white geese (Anser cygnoides), and Hungarian geese (Anser anser). Poult Sci.

[15] Ibrahim D, Goshu G, Esatu W, Cahaner A (2019). Dual-purpose production of genetically different chicken crossbreeds in Ethiopia. 2. Egg and meat production of the final-crossbreed females and males. Poult Sci.

[16] Wang HH, Cahaner A, Lou LF (2022). Genetics and breeding of a black-bone and blue eggshell chicken line. 2. Laying patterns and egg production in two consecutive generations. Poult Sci.

[17] Ministry of Agriculture (2004). Feeding standard of chicken (NY/T33-2004).

[18] Akil R, Zakaria AH (2015). Egg laying characteristics, egg weight, embryo development, hatching weight and post-hatch growth in relation to oviposition time of broiler breeders. Anim Reprod Sci.

[19] Alders R, Tomley F (2022). Animal board invited opinion paper: planet, people and poultry - more and better data needed to get the balance right. Animal.

[20] Isa AM, Sun YY, Li YL (2022). MicroRNAs with non-additive expression in the ovary of hybrid hens target genes enriched in key reproductive pathways that may influence heterosis for egg laying traits. Front Genet.

[21] Wang YM, Yuan JW, Sun YY (2022). Genetic basis of sexual maturation heterosis: insights from ovary lncRNA and mRNA repertoire in chicken. Front Endocrinol.

[22] Wang YM, Sun YY, Ni AX (2022). Research Note: Heterosis for egg production and oviposition pattern in reciprocal crossbreeds of indigenous and elite laying chickens. Poult Sci.

[23] Wolc A, Bednarczyk M, Lisowski M, Szwaczkowski T (2010). Genetic relationships among time of egg formation, clutch traits and traditional selection traits in laying hens. J Anim Feed Sci.

[24] Renema RA, Robinson FE, Zuidhof MJ (2007). Reproductive efficiency and metabolism of female broiler breeders as affected by genotype, feed allocation, and age at photostimulation. 2. Sexual maturation. Poult Sci.

[25] Zuidhof MJ, Renema RA, Robinson FE (2007). Reproductive efficiency and metabolism of female broiler breeders as affected by genotype, feed allocation, and age at photostimulation. 3. Reproductive efficiency. Poult Sci.

[26] Shi L, Sun Y, Xu H (2019). Effect of age at photostimulation on reproductive performance of Beijing-You Chicken breeders. Poult Sci.

[27] Samiullah S, Roberts J, Chousalkar K (2016). Oviposition time, flock age, and egg position in clutch in relation to brown eggshell color in laying hens. Poult Sci.

